# Supplementary material for: Heavy Load Carrying and Symptoms of Pelvic Organ Prolapse among Women in Tanzania and Nepal: An Exploratory Study
Source: Int J Environ Res Public Health. 2021 Jan 31;18(3):1279. doi: 10.3390/ijerph18031279 (PMC7908160; doi:10.3390/ijerph18031279)
Supplement: Supplementary file 1 [file ijerph-18-01279-s001.pdf]

Kirk Center for Healthy Living
Therapy for Pelvic Pain, Incontinence, Infertility and Gastrointestinal Disorders

16618 W 159th St, Suite 401 & 402, Lockport, IL 60441
Phone: **(815) 838-0529** ❖ Fax: **(815) 838-0652**
Website: **kc4hl.com**

Name: ______________________________ **Date:** ______________________

PFDI-20 Instructions: Please answer all of the questions in the following survey. These questions will ask you if you have certain bowel, bladder, or pelvic symptoms and, if you do, how much they bother you. Answer these by circling the appropriate number. While answering these questions, please consider your symptoms over the **last 3 months**.

The PFDI-20 has 20 items and 3 scales of your symptoms. All items use the following format with a response scale from 0 to 4.

Symptoms Present = YES: 1 = not at all, 2 = somewhat, 3 = moderately, 4 = quite a bit
Not Present = NO: 0 = not present

Pelvic Organ Prolapse Distress Inventory 6 (POPDI-6):

Do you….	**No**	**Yes**
1. Usually experience pressure in the lower abdomen?	0	1 2 3 4
2. Usually experience heaviness or dullness in the pelvic area?	0	1 2 3 4
3. Usually have a bulge or something falling out that you can see or feel in your vaginal area?	0	1 2 3 4
4. Ever have to push on the vagina or around the rectum to have or complete a bowel movement?	0	1 2 3 4
5. Usually experience a feeling of incomplete bladder emptying?	0	1 2 3 4
6. Ever have to push up on a bulge in the vaginal area with your fingers to start or complete urination?	0	1 2 3 4

Colorectal-Anal Distress Inventory 8 (CRAD-8):

Do you….	**No**	**Yes**
7. Feel you need to strain too hard to have a bowel movement?	0	1 2 3 4
8. Feel you have not completely emptied your bowels at the end of a bowel movement?	0	1 2 3 4
9. Usually lose stool beyond your control if your stool is well formed?	0	1 2 3 4
10. Usually lose stool beyond your control if your stool is loose?	0	1 2 3 4
11. Usually lose gas from the rectum beyond your control?	0	1 2 3 4
12. Usually have pain when you pass your stool?	0	1 2 3 4
13. Experience a strong sense of urgency and have to rush to the bathroom to have a bowel movement?	0	1 2 3 4
14. Does part of your bowel ever pass through the rectum and bulge outside during or after a bowel movement?	0	1 2 3 4

Colorectal-Anal Distress Inventory 8 (CRAD-8):

Do you….	**No**	**Yes**
15. Usually experience frequent urination?	0	1 2 3 4
16. Usually experience urine leakage associated with it feeling of urgency, that is, a strong sensation of needing to go to the bathroom?	0	1 2 3 4
17. Usually experience urine leakage related to coughing, sneezing, or laughing?	0	1 2 3 4
18. Usually experience small amounts of urine leakage (that is, drops)?	0	1 2 3 4
19. Usually experience difficulty emptying your bladder?	0	1 2 3 4
20. Usually experience *pain* or *discomfort* in the lower abdomen or genital region?	0	1 2 3 4

Scoring the PFDI.20:

Scale Scores: Obtain the mean value of all of the answered Items within the corresponding scale (possible value 0 to 4) and then multiply by 25 to obtain the scale score (range 0 to 100). Missing items are dealt with by using the mean from answered items only.

PFSI-20 Summary Score: Add the scores from the 3 scales together to obtain the summary score (range 0 to 300).
